# Community Composition of Nitrous Oxide Consuming Bacteria in the Oxygen Minimum Zone of the Eastern Tropical South Pacific

**DOI:** 10.3389/fmicb.2017.01183

**Published:** 2017-06-28

**Authors:** Xin Sun, Amal Jayakumar, Bess B. Ward

**Affiliations:** Department of Geosciences, Princeton University, PrincetonNJ, United States

**Keywords:** N_2_O consuming bacteria, *nosZ* gene, microarray, oxygen minimum zone, Eastern Tropical South Pacific

## Abstract

The ozone-depleting and greenhouse gas, nitrous oxide (N_2_O), is mainly consumed by the microbially mediated anaerobic process, denitrification. N_2_O consumption is the last step in canonical denitrification, and is also the least O_2_ tolerant step. Community composition of total and active N_2_O consuming bacteria was analyzed based on total (DNA) and transcriptionally active (RNA) nitrous oxide reductase (*nosZ*) genes using a functional gene microarray. The total and active *nosZ* communities were dominated by a limited number of *nosZ* archetypes, affiliated with bacteria from marine, soil and marsh environments. In addition to *nosZ* genes related to those of known marine denitrifiers, atypical *nosZ* genes, related to those of soil bacteria that do not possess a complete denitrification pathway, were also detected, especially in surface waters. The community composition of the total *nosZ* assemblage was significantly different from the active assemblage. The community composition of the total *nosZ* assemblage was significantly different between coastal and off-shore stations. The low oxygen assemblages from both stations were similar to each other, while the higher oxygen assemblages were more variable. Community composition of the active *nosZ* assemblage was also significantly different between stations, and varied with N_2_O concentration but not O_2_. Notably, *nosZ* assemblages were not only present but also active in oxygenated seawater: the abundance of total and active *nosZ* bacteria from oxygenated surface water (indicated by *nosZ* gene copy number) was similar to or even larger than in anoxic waters, implying the potential for N_2_O consumption even in the oxygenated surface water.

## Introduction

N_2_O is a major ozone-depleting substance and a greenhouse gas whose radiative forcing per mole is 298 times that of carbon dioxide (IPCC, 2007; Ravishankara et al., 2009). Oxygen minimum zones (OMZs) are the most intense marine sources of N_2_O and are hot spots of rapid N_2_O cycling (Martinez-Rey et al., 2015). OMZs are marine regions with a strong O_2_ gradient (oxycline) overlying an oxygen deficient zone (ODZ) where O_2_ concentration is low enough to induce anaerobic processes. The global expansion and intensification of OMZs, which are predicted to result from global warming, further stress the importance of understanding N_2_O cycling in these regions (Codispoti, 2010). N_2_O production and consumption are driven by marine bacteria (Naqvi et al., 2000). The dominant microbial process for N_2_O cycling is denitrification, the sequential reduction of NO_3_^-^ to NO_2_^-^, NO, N_2_O and finally to N_2_ (Zumft, 1997; Naqvi et al., 2000). Denitrification could stop at an intermediate step before N_2_ if O_2_ concentration exceeds the threshold for the latter step or if electron donors are depleted (Ward et al., 2008; Dalsgaard et al., 2012; Babbin et al., 2014). Thus O_2_ concentration or electron donor availability could also control the N_2_O budget. N_2_O concentrations and net N_2_O production rates (N_2_O production minus N_2_O consumption) were found to peak at the oxic-suboxic interface in OMZs, due to excess production from nitrification and incomplete denitrification (Nicholls et al., 2007; Ji et al., 2015; Trimmer et al., 2016). However, while multiple processes can produce N_2_O, reduction by N_2_O consuming bacteria is the only known biological N_2_O sink.

N_2_O consumption is the final step of denitrification, and is the least O_2_ tolerant step (Bonin et al., 1989; Körner and Zumft, 1989). N_2_O consumption rates have been measured in ODZs at depths where O_2_ concentration ranged from very low to below the detection limit (Wyman et al., 2013; Babbin et al., 2015). N_2_O consumption by denitrification and genes involved in N_2_O reduction have also been detected in oxygenated seawater (Farías et al., 2009; Wyman et al., 2013). Characterizing the distribution and environmental regulation of this step is necessary for a complete quantification of the oceanic N_2_O budget and will improve our ability to predict oceanic N_2_O emissions under global climate change.

N_2_O consumption is catalyzed by the enzyme nitrous oxide reductase, encoded by the *nosZ* gene. A recent study of *nosZ* genes found a lower diversity of *nosZ* genes in ODZ waters than in the upper oxycline of the OMZ (Castro-González et al., 2015). The distribution of *nosZ* genes was related to O_2_ concentration, which suggested that the quantity and composition of *nosZ* genes and the diversity of denitrifying bacteria might influence the microbial potential for N_2_O consumption.

We aimed to determine the distribution and community composition of total and transcriptionally active (abbreviated as ‘active’ hereafter) *nosZ* assemblages based on the presence (DNA) and expression (RNA) of *nosZ* genes in the OMZ of the Eastern Tropical South Pacific (ETSP), one of the three major OMZs in the world ocean. Three hypotheses were tested in the study: (1) the community compositions of total and active *nosZ* assemblages differ between coastal and off-shore stations because the quantity and quality of the nutrients at the two stations differ due to different contributions from land and sediment; (2) quantities and composition of total and active *nosZ* assemblages are related to O_2_ concentration because N_2_O consumption is the least O_2_ tolerant step in conventional denitrification; and (3) the distribution of the active *nosZ* assemblage is more related to N_2_O concentration than that of total *nosZ* assemblage because the former indicates live and active organisms.

## Materials and Methods

### Experimental Sites and Sampling

Samples were collected on the *R/V Nathaniel B. Palmer* during June to July 2013 (cruise NBP 1305) in the OMZ of the ETSP at the off-shore station (BB1; 14.0°S, 81.2°W) and the coastal station (BB2; 20.50°S, 70.70°W) (Supplementary Figure 1). Particulate material was collected in Niskin bottles mounted on the standard conductivity-temperature-depth (CTD) rosette system (Seabird Electronics, Seattle, WA, United States) at four depths at each station (BB1: 60, 130, 300, and 1000 m; BB2: 60, 115, 300, and 1000 m) and concentrated by filtration (up to 4 L) through Sterivex filters (0.22 μm). Filters were flash frozen in liquid nitrogen onboard and stored at -80°C until DNA and RNA extraction was performed.

Temperature, salinity, sigma theta, bottom depth and pressure at each station were measured on the SBE 911+ CTD system. Fluorescence, representing chlorophyll a, was measured using a single channel fluorometer (Wet labs, Philomath, OR, United States) mounted on the CTD. Oxygen distributions were determined using the STOX sensor (detection limit = 10 nM) mounted on the CTD rosette (Revsbech et al., 2009). Ammonium, nitrite and nitrate concentrations were measured using standard colorimetric protocols (UNESCO, 1994). N_2_O concentration was determined using mass spectrometry (Ji et al., 2015). N^∗^ is the deviation of measured dissolved inorganic nitrogen (DIN = nitrate + nitrite + ammonia) from predicted DIN by Redfield ratio and the world-ocean nitrogen to phosphate regression relationship (Deutsch et al., 2001). Environmental data were reported by Ji et al. (2015) and are provided in Supplementary Table 1.

### DNA and RNA Extractions

Both DNA and RNA were extracted from eight Sterivex filters using the plant tissue protocol of the All Prep DNA/RNA Mini Kit (50) using a QIAcube (Qiagen). Reverse transcription from RNA to cDNA was performed using SuperScript^®^ III First-Strand Synthesis System for RT-PCR (Invitrogen^TM^ by Life Technologies^TM^). Excess RNA was removed by RNase at the end of the synthesis.

### Quantitative PCR Assays

The abundance of total and active *nosZ* assemblages were estimated by quantitative PCR (qPCR) using SYBR^®^ Green based assays using protocols described previously (Jayakumar et al., 2013). Primers nosZ1F and nosZ1R (Henry et al., 2006) were used to amplify a 259-bp conserved fragment of the *nosZ* gene. Known quantities (∼20–25 ng) of DNA and cDNA samples were assayed along with a minimum of five serial dilutions of plasmids containing *nosZ* gene, no template controls and no primer controls, all in triplicate on the same plate. To maintain continuity and consistency among qPCR assays, a subset of samples from the first qPCR assay was run with subsequent assays and fresh standard dilutions were prepared for each assay. DNA, cDNA and the concentrated standards were quantified prior to every assay using PicoGreen fluorescence (Molecular Probes, Eugene, OR, United States) calibrated with several dilutions of phage lambda standards, to account for DNA loss due to freeze thaw cycles. qPCR assays were run on a Stratagene MX3000P (Agilent Technologies, La Jolla, CA, United States). Automatic analysis settings were used to determine the threshold cycle (*C*t) values. The copy numbers (number of copies of the gene sequence detected in the sample) were calculated according to: *Copy number* = (*ng ^∗^ number/mole*)/(*bp ^∗^ ng/g ^∗^ g/mole of bp*) and then converted to copy number per ml seawater filtered, assuming 100% extraction efficiency.

### Microarray Experiments

DNA and cDNA qPCR products were used as targets for microarray experiments to characterize the community composition of total and active *nosZ* assemblages, respectively. Triplicate qPCR products from each depth were pooled. *nosZ* gene targets were purified and extracted from agarose gels using the QIAquick gel extraction kit (Qiagen). Purified DNA qPCR products from eight depths and cDNA qPCR products from seven depths were used to prepare targets for microarray analysis.

Microarray targets were prepared from the qPCR products following the protocol of Ward and Bouskill (2011). Briefly, dUaa was incorporated into purified DNA and cDNA during linear amplification using the BioPrime kit (Invitrogen^TM^). The dUaa-Klenow product was labeled with Cy3 (dissolved in dimethyl sulfoxide), purified using QIAquick columns (Qiagen) and quantified by Nanodrop 2000 (Thermo Scientific). Duplicate Cy3 products for each sample were hybridized at 65°C overnight (16 h) onto replicate microarrays under ozone free conditions. Hybridized microarrays were washed and scanned with an Axon 4300 laser scanner.

### ***nosZ*** Microarray

The microarray (BC016) contains 114 *nosZ* archetype probes. Each probe is a 90-bp sequence comprised of a 70-bp *nosZ* gene fragment and a 20-bp control region. Each archetype probe represents, and hybridizes with, all *nosZ* sequences with >85% identity, based on published sequences available in 2013. There are 71 NosZ archetypes, which represent typical or Clade I *nosZ* genes, and 43 WNZ archetypes, which represent the atypical or Clade II *nosZ* genes. The development of the microarray is described in Jayakumar et al. (in preparation) and the sequences are shown in Supplementary Table 2.

### Data Analyses

Fluorescence signal intensities for *nosZ* probes hybridized to the microarrays were obtained using GenePix Pro 7 software. The fluorescence ratio (FR) of each feature is defined as the ratio Cy3/Cy5 (70-mer probe/20-mer standard for each feature). The FR for each *nosZ* archetype was calculated as the average of probe signal intensities for duplicate features on the same microarray. Normalized fluorescence ratio (FRn) was calculated by dividing the FR of each *nosZ* probe by the maximum *nosZ* FR on the same microarray. FRn is the proxy of the relative abundance of each archetype and was used for further analyses.

Detrended correspondence analysis (DCA) was performed to analyze the overall microbial community composition. A dissimilarity test was performed using Permutational Multivariate Analysis of Variance (*adonis*). α-diversities (Shannon diversity indices) of total and active *nosZ* assemblages were calculated. β-diversities (Bray–Curtis dissimilarities) of total and active *nosZ* assemblages between different sites (i.e., depths) were calculated to perform a Mantel test. The Mantel test was used to determine significant environmental variables correlated with microbial community composition. These analyses were carried out using the vegan package in R (version 3.3.1). A maximum likelihood phylogenetic tree was built from aligned archetype sequences with MEGA 7 software. FRn values for each archetype at different depths from both stations were visualized on the phylogenetic tree by iTOL^1^. The copy number of *nosZ* genes at each depth is given as mean (± standard error) of the qPCR triplicates.

## Results

### Abundance and Depth Distribution of Total and Active ***nosZ*** Assemblages

**FIGURE 1:**
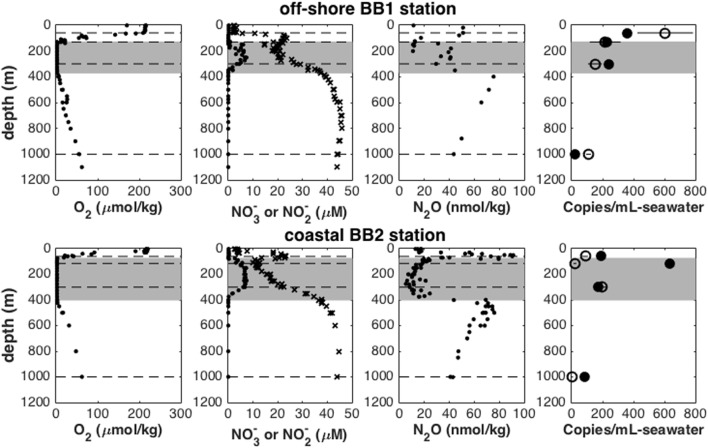
O_2_, NO_3_^-^ (x), NO_2_^-^, N_2_O and copy number of total (DNA, filled circles) and active (RNA, open circles) profiles at off-shore station BB1 and coastal station BB2. Dashed lines indicate depths where samples were collected. Gray areas indicate ODZs.

At stations BB1 and BB2, the continuously undetectable O_2_ concentration, the local nitrite maximum and the nitrate deficit at intermediate depths (130–370 m at BB1; 75–400 m at BB2) all indicated the presence of ODZs (gray areas in **Figure 1**). Sampling depths were chosen to represent water column features defined by oxygen concentration, as measured with the *in situ* STOX sensor: oxygenated surface water, upper oxycline [characterized by sharp O_2_ concentration gradient ranging from saturation to below detection limit (<10 nM)], top of the ODZ (O_2_ concentration <10 nM), core of the ODZ (O_2_ concentration <10 nM) and lower oxycline (O_2_ concentration >10 nM). The abundance of the total *nosZ* genes ranged from 24.1 (±1.4) copies mL^-1^ in a sample from the lower oxycline to 636.4 (±28.3) copies mL^-1^ in a sample from the ODZ (**Figure 1**). As for the active *nosZ* assemblage, the lowest abundance of active *nosZ* genes was 5.1 (±0.5) copies mL^-1^ in a sample from the lower oxycline and the highest abundance was 604.6 (±103.7) copies mL^-1^ in a sample from the surface water.

The abundance of total and active *nosZ* assemblages showed different distribution patterns at the two stations (**Figure 1**). At station BB1, the abundance of both total and active *nosZ* genes was highest in a sample from the surface water, and decreased with depth. At station BB2, the abundance of both total and active *nosZ* genes peaked in samples from the ODZ and was lowest in samples from the lower oxycline. The active *nosZ* genes were most abundant in the sample from 300 m. However, the total *nosZ* genes were most abundant in the sample from 115 m, where the abundance of the active *nosZ* genes was only 3% of the total.

### Diversity and Dominant Archetypes of Total and Active ***nosZ*** Assemblages

**FIGURE 2:**
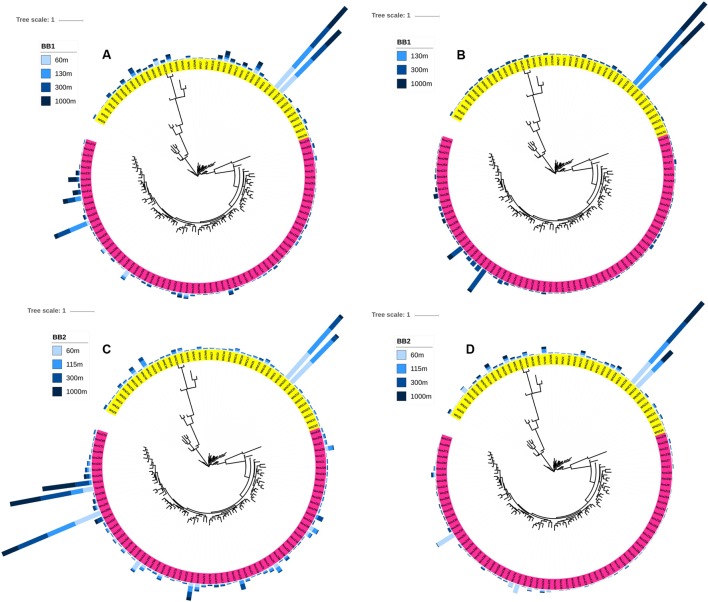
Maximum likelihood trees for **(A,C)** total and **(B,D)** active *nosZ* archetypes at off-shore station BB1 **(A,B)** and coastal station BB2 **(C,D)**. Pink = typical *nosZ* clade, yellow = atypical *nosZ* clade. Bars in outer cycles stand for mean FRn of duplicates of each archetype.

The distribution of FRn of the total or active archetypes was similar across all depths within the same station (**Figure 2**). The average α-diversity was not significantly different (student’s *t*-test, *P* = 0.102) between the total assemblages (3.21) and the active assemblages (2.60) (**Table 1**). The least diverse total assemblage was from the lower oxycline (1000 m of station BB2), but the two least diverse active assemblages were from the ODZs (130 m of BB1 and 300 m of BB2).

**Table 1:** α-diversities of total (DNA) and active (RNA) *nosZ* genes at off-shore station BB1 and coastal station BB2.

	Depth(m)	DNA	RNA		Depth(m)	DNA	RNA
Off-shore station BB1	60	3.18		Coastal station BB2	60	3.33	3.39
	130	3.09	1.29		115	3.56	2.75
	300	3.57	3.37		300	3.57	1.63
	1000	3.29	2.56		1000	2.10	3.19


**FIGURE 3:**
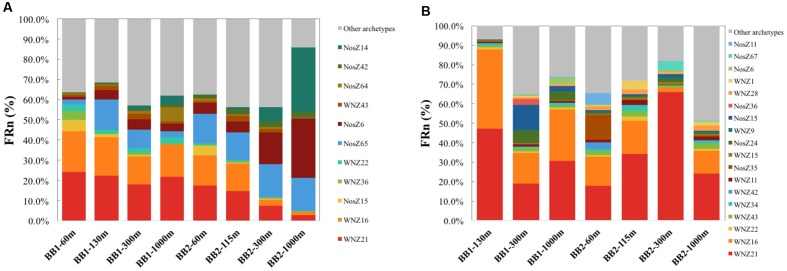
**(A)** total (DNA) and **(B)** active (RNA) *nosZ* archetypes whose FRn signals are among the top five highest in any of the depths at off-shore station BB1 and coastal station BB2.

The FRn distribution of *nosZ* archetypes showed that a very limited number of archetypes dominated the total or the active *nosZ* assemblages (**Figure 2**). Dominant archetypes were affiliated with bacteria from various environments, including salt marsh, soil, marine sediment, marine hot spring and activated sludge of a wastewater treatment plant (Supplementary Tables 3, 4). The FRn of the top five most abundant archetypes accounted for 48.9 to 83.3% of the total *nosZ* hybridization signal (**Figure 3A** and Supplementary Table 3). Notably, the highest percentage (83.3%) was from the sample from the lower oxycline (1000 m) at station BB2 and the most abundant archetype (NosZ42, an uncultured clone of *nosZ* gene derived from salt marsh sediments; Kearns et al., 2015) accounted for 31.6% of total FRn. However, this archetype was not among the top five archetypes of the active *nosZ* assemblage in the same sample (**Figure 3B** and Supplementary Table 4). The two most dominant typical *nosZ* archetypes in the total assemblage were NosZ6, derived from an uncultured clone from salt marsh sediments (Kearns et al., 2015), which is closely related to *Marinobacter*, and NosZ65, derived from *Marinobacter* sp. BSs20148 from marine sediment (Song et al., 2013) (**Figure 3A** and Supplementary Table 3). In contrast, NosZ6 and NosZ65 were not dominant in the active assemblage (**Figure 3B** and Supplementary Table 4).

The sample from the ODZ (130 m) at station BB1 illustrates the contrasts observed between total and active *nosZ* assemblages. The FRns of the top three dominant archetypes (WNZ21, WNZ16 and NosZ65) in the total *nosZ* assemblage were comparable to each other, and they constituted 56.1% of the total community (Supplementary Table 3). However, NosZ65 was nearly undetectable in the active assemblage and the top two dominant archetypes (WNZ21 and WNZ16) accounted for 87.8% of the active assemblage in the same sample (Supplementary Table 4). The total *nosZ* assemblage was much more diverse than the active assemblage at this depth (**Table 1**). The representative sequences of WNZ21 and WNZ16 archetypes were derived from *nosZ* gene sequences of *Anaeromyxobacter dehalogenans* strain DCP18 (Chee-Sanford et al., unpublished) and an uncultured bacterium clone obtained from agricultural soils (Sanford et al., 2012), respectively. WNZ21 and WNZ16 archetypes were not only dominant in the active assemblage in one sample from the ODZ, but were among the top five abundant archetypes of both total and active assemblages in almost all samples (**Figure 3**).

### Community Composition of Total and Active ***nosZ*** Assemblages

**FIGURE 4:**
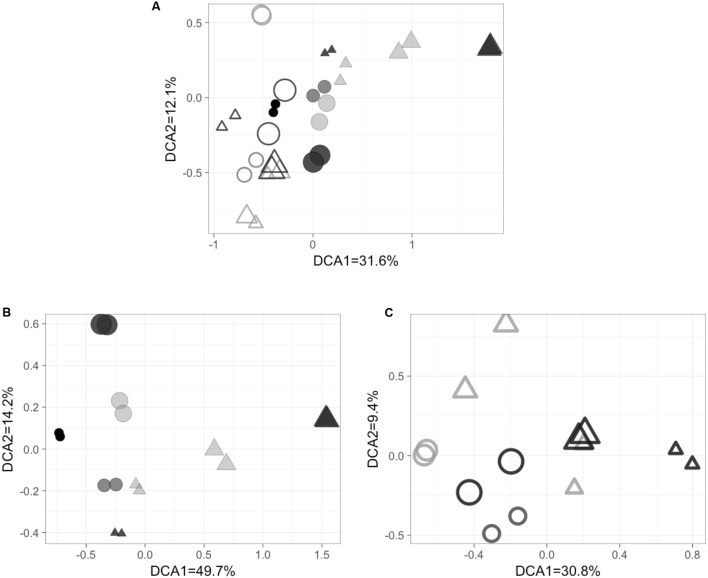
Detrended correspondence analysis (DCA) results of **(A)** total and active *nosZ* genes, **(B)** the total genes only and **(C)** the active genes only. Filled symbol stands for total *nosZ* genes, open symbol stands for active *nosZ* genes. Circles = off-shore station BB1 and triangles = coastal station BB2. The symbol size represents depths (the larger, the deeper). Symbol color represents O_2_ concentration (the darker, the higher). Symbols with the same shape, color and size were duplicates. Three pairs of symbols in lightest gray in **(B)** indicates samples from the ODZ with O_2_ concentration under detection limit.

Functional gene microarrays were used to describe the community composition of *nosZ* assemblages. FRn values from duplicate microarrays replicated well (*r*^2^ = 0.802–0.997) (Supplementary Figure 2) and each pair of duplicates clustered together in the DCA plots (**Figure 4**).

The two-dimensional DCA model including both DNA and RNA microarray results explained 43.7% of the community composition of *nosZ* assemblages with 31.6% explained by the first axis and 12.1% explained by the second axis (**Figure 4A**). The clearest pattern was the clear separation of total (filled symbols) and active (open symbols) *nosZ* assemblages, indicating that they were different from each other. The significance (*P* < 0.001) of the difference between total and active assemblages was confirmed by the dissimilarity test (**Table 2**). Therefore, the community composition of total and active *nosZ* assemblages was further analyzed by two DCA models separately to better examine other patterns.

**Table 2:** *P*-values of dissimilarity test (*adonis*).

	DNA and RNA samples	DNA samples only	RNA samples only
DNA versus RNA	<0.001	—	—
Site dependence	0.034	<0.001	0.039


The DCA model of DNA microarray results explained 63.9% of the composition of the total *nosZ* assemblage (**Figure 4B**). The total *nosZ* community composition revealed site difference and O_2_ dependence. Samples from the same station clustered together in the DCA model, indicating community composition was different between the off-shore station (BB1) and the coastal station (BB2). The site difference was statistically significant (*P* < 0.001) based on the dissimilarity test (**Table 2**). Besides the geographical pattern, composition of the total *nosZ* assemblage was also affected by O_2_ concentration (**Figure 4B**). Samples from the ODZs clustered together, while samples with higher O_2_ concentrations were distinct from the ODZ samples and different from each other. O_2_ concentration of the seawater might not be the most important driver of microbial community composition, however, since the O_2_ pattern was not captured by either axis of the DCA model.

The DCA model of RNA microarray results explained 40.2% of the community composition of the active *nosZ* assemblage (**Figure 4C**). Significant site difference (*P* = 0.039) of the active community composition was also revealed by the DCA model (**Figure 4C**) and the dissimilarity test (**Table 2**). However, the clustering based on O_2_ concentration that was observed in the total *nosZ* assemblage was not observed for the active *nosZ* assemblage.

### Environmental Variables Correlated with the Community Composition

The composition of total and active *nosZ* assemblages was correlated with different environmental variables based on a Mantel test (**Table 3**). Relative depth, nitrate concentration, temperature, density (sigma theta) and pressure were significantly related to the β-diversity of the total *nosZ* assemblage. However, the β-diversity of the active *nosZ* assemblage was significantly related to N_2_O concentration, nitrite concentration and fluorescence. Bottom depth, which was dramatically different between two stations and is a proxy for important ecological differences between the two sites, was a significant factor for both total and active *nosZ* assemblages.

**Table 3:** Mantel tests between total (DNA) or active (RNA) *nosZ* genes and environmental factors.

	Total *nosZ*		Active *nosZ*	
		
	*r*	*P*	*r*	*P*
**Oxygen**	0.169	0.190	-0.046	0.636
**Relative Depth^1^**	0.848	**0.001**	-0.170	0.886
**Nitrate**	0.355	**0.009**	-0.149	0.913
**Nitrite**	0.090	0.178	0.300	**0.019**
**Nitrous Oxide**	-0.089	0.684	0.415	**0.014**
**Temperature**	0.483	**0.002**	-0.153	0.937
**Ammonium**	0.155	0.234	-0.008	0.458
**Salinity**	0.258	0.069	-0.165	0.941
**Sigma Theta^2^**	0.439	**0.007**	-0.088	0.774
**Bottom Depth**	0.320	**0.001**	0.255	**0.012**
**Pressure**	0.492	**0.003**	-0.168	0.949
**Fluorescence**	0.059	0.290	0.320	**0.015**


*^1^Relative depth was calculated by dividing measured depth by the bottom depth of each station. ^2^Sigma Theta was density calculated with *in situ* salinity and potential temperature at zero pressure. Bolded P values indicate significant correlation (P < 0.05).*

## Discussion

### Abundance and Diversity of ***nosZ*** Assemblages

Oxygen minimum zones are sites of high N_2_O flux to the atmosphere (Law and Owens, 1990; Arévalo-Martínez et al., 2015). N_2_O consuming organisms are the only biological sink for N_2_O. Hence their abundance and community composition in the OMZ may be important in understanding the N_2_O flux. The abundance of total and active N_2_O consuming bacteria in the OMZ of the ETSP was estimated by measuring *nosZ* gene copy number (**Figure 1**). The relationship between abundance of N_2_O consuming bacteria and depth in this study differed from that of denitrifiers indicated by *nirS* gene copy number at the same stations (Ji et al., 2015): the abundances of the total and active N_2_O consuming bacteria in the surface water were similar to or higher than those in the ODZs (**Figure 1**), but the abundance of denitrifiers in the surface water was two orders of magnitude smaller than that in the ODZs. This difference suggests that the two genes represent functionally different groups.

*nirS* and *nosZ* also differed in their absolute abundance. The highest abundance of N_2_O consuming bacteria was only a few hundred copies mL^-1^, which was three orders of magnitude smaller than the highest abundance of denitrifiers measured at the same stations (Ji et al., 2015). It is assumed that both genes are present in the genome as single copy genes, although there are exceptions for *nosZ* (Sanford et al., 2012). One possible explanation for the differences in both distribution and abundance of the *nosZ* assemblage and the *nirS* assemblage is that not all N_2_O consuming bacteria contain the complete denitrification gene sequence (Sanford et al., 2012). The atypical *nosZ* genes are associated with bacteria that lack the other steps in the conventional denitrification pathway. Notably, bacteria with only *nosZ* genes but no other denitrification genes were overrepresented in the genomes of marine bacteria compared to other ecosystems (Graf et al., 2014). *nirS*, however, was preferentially associated with bacteria that contained a complete denitrification pathway (Graf et al., 2014).

Another contributing factor may be the specificity or bias of the PCR primers. The *nosZ* primers used in this study were optimized to amplify all known *nosZ* sequences as of 2006, and should therefore represent the large database of both terrestrial and marine sequences available at the time. However, it is clear that they might underrepresent the atypical N_2_O consuming bacteria, which were not known at the time. The *nirS* primers used in the previous analysis of these samples (Ji et al., 2015) are potentially biased toward marine sequences (Braker et al., 1998) and may underrepresent more diverse sequences now available from other environments. One way to improve the *nosZ* coverage is to use multiple primer sets targeting different groups of *nosZ* archetypes.

The N_2_O consuming bacteria are a small component of the total microbial assemblage, but are still quite diverse (Jones et al., 2013), so they are difficult to characterize by pure culture or metagenomics. The microarray, which was designed specifically to target N_2_O consuming bacteria using more than 100 *nosZ* gene probes (**Figure 2**), may be a better tool to capture these underrepresented organisms without cultivation or detection of rare sequences in complex metagenomic datasets. The high reproducibility of microarrays reported previously (Bulow et al., 2008) was confirmed in this study in that duplicates for each sample run on two different microarrays clustered together in DCA (**Figure 4**) and had high *r*^2^ of linear regressions (Supplementary Figure 2).

Based on the FRn values of diverse *nosZ* archetypes determined by microarray hybridization (**Figure 2**), a very limited number of archetypes dominated the total or the active assemblages. Moreover, the top five active archetypes accounted for larger percentage of the assemblage than that of the total archetypes (**Figure 3**), consistent with the less diverse active assemblage compared to the total assemblage (**Table 1**). These findings imply that although the total *nosZ* assemblage is very diverse, a few *nosZ* archetypes might be the major contributors of N_2_O consumption at the study sites. The relative abundance of active archetypes, however, might uncouple that of enzymes of different archetypes and/or the contribution of different archetypes to the N_2_O consumption rate due to different stabilities of enzymes from different archetypes.

### Total and Active Community Compositions and Their Controlling Environmental Variables

The active *nosZ* assemblage was different from the total assemblage in both abundance profiles and community composition as detected by qPCR and *nosZ* microarray hybridization analysis, respectively. The highest abundance of the total N_2_O consuming bacteria indicated by *nosZ* DNA copy number was 636.4 (±28.3) copies mL^-1^ in the sample from the ODZ (115 m) at station BB2, but the abundance of the active bacteria in the same sample was only 21.1 (±5.2) copies mL^-1^ (**Figure 1**). In the sample from oxygenated surface seawater (60 m) at station BB1, the abundance of the active N_2_O consuming bacteria was 604.6 (±103.7) copies mL^-1^, but the abundance of the total bacteria was only 357.2 (±12.5) copies mL^-1^ (**Figure 1**).

Significant differences between the community composition of total and active N_2_O consuming bacteria were indicated by the results of DCA and dissimilarity test (**Figure 4A**; **Table 2**). The different community composition was attributed to the differences in the distribution of FRn of more than 100 archetypes, especially the dominant ones. NosZ6 and NosZ65 were dominant in the total *nosZ* assemblage but were minor components in the active assemblage. NosZ42 was the most abundant archetype in the total *nosZ* assemblage from the lower oxycline (1000 m) at station BB2, but was not among the top five archetypes of the active assemblage. These differences between active and total *nosZ* communities are consistent with observations from soil and salt marsh sediments in which the active component of the microbial assemblage was apparently more responsive to environmental conditions (Barnard et al., 2013; Kearns et al., 2016).

Despite the differences between the total and the active N_2_O consuming assemblages, their distributions both depended on geographic location. The composition of total and active *nosZ* assemblages was significantly different between the coastal and the off-shore stations as indicated by the results of DCA and dissimilarity test. Since the two stations shared similar dominant *nosZ* archetypes, the geographical divergence mainly reflected differences among the large number of rare archetypes between the two stations. The more negative N^∗^ at the coastal station BB2 (Supplementary Table 1) indicates more intense nitrogen-loss fueled by more organic matter. The different amount of organic matter, which supports the metabolism of heterotrophic *nosZ* bacteria, might partially contribute to the geographical differences of *nosZ* assemblages. Geographical differences might also result from different nutrient sources at the two stations, since their distance to the sediment (bottom depth) and to the shore were dramatically different. The dependence of geographic location was also observed for ammonia oxidizing archaea (Peng et al., 2013) and for *nirS* denitrifiers (Jayakumar et al., 2013) in the ETSP and Arabian Sea OMZs.

In addition to geographical patterns, the total and active assemblages were correlated with different environmental variables (**Table 3**). Depth and environmental parameters that co-varied with depth (including temperature, density and pressure) were major drivers of the β-diversity of the total N_2_O consuming assemblages, implying different organisms coexist in the water column by occupying different ecological niches. However, N_2_O concentration difference was a major driver of the β-diversity of the active *nosZ* assemblages, implying the active *nosZ* community was a better indicator for N_2_O consumption potential.

### ***nosZ*** Assemblage in Oxygenated Seawater

The role of the *nosZ* assemblage in oxygenated seawater has been ignored because N_2_O consumption is considered the least oxygen tolerant anaerobic step in the conventional denitrification pathway (Zumft, 1997). However, *nosZ* genes were abundant in oxygenated surface water in the Southern Indian Ocean (Raes et al., 2016) and *nosZ* mRNAs were detected in the oxic regions in the Arabian Sea (Wyman et al., 2013). Our study confirmed that a *nosZ* assemblage was not only present but also active in oxygenated surface water in the OMZ of the ETSP. In particular, atypical *nosZ* archetypes, usually associated with N_2_O consuming bacteria lacking a complete denitrification pathway, were present and active in surface waters. In addition, the most abundant archetypes of total and active *nosZ* communities were both atypical *nosZ* archetypes (WNZ21 and WNZ16), implying the significant contribution of atypical archetypes to the *nosZ* communities and the necessity to consider atypical archetypes while analyzing the potential of N_2_O consumption.

N_2_O reductase enzymes from denitrifiers had very low O_2_ tolerance (Bonin et al., 1989; Körner and Zumft, 1989); on the contrary, *nosZ* assemblages were detected in the oxygenated surface waters and O_2_ concentration was not significantly correlated with the active microbial community, as indicated by DCA and Mantel test. The survival of N_2_O consuming bacteria in oxic layers and their O_2_-independence might be attributed to anoxic micro-environments created by phytoplankton microaggregates or particles. Free-living and particle-associated microbes from the same seawater sample can have different community compositions (Delong et al., 1993). More specifically, a recent study in the OMZ of the ETSP showed that *nosZ* mRNAs were 28-fold more abundant on particles (>1.6 μm) compared to free-living microbes (0.2–1.6 μm) (Ganesh et al., 2015). Additionally, *nosZ* mRNA co-occurred with the cyanobacterium *Trichodesmium* in oxic water in the Arabian Sea (Wyman et al., 2013). Consistently, fluorescence, a proxy for chlorophyll a, was significantly correlated with the β-diversity of the active *nosZ* assemblages in this study (**Table 3**).

The active *nosZ* community in oxygenated surface water might capture N_2_O produced in deeper seawater and thus reduce the flux into the atmosphere. Thus, evaluating the *nosZ* community is essential to the prediction of the oceanic N_2_O emissions. Moreover, the oceanic N_2_O emissions represent net fluxes, which are controlled by both N_2_O production and N_2_O consumption. Some N_2_O flux models (Suntharalingam and Sarmiento, 2000; Martinez-Rey et al., 2015; Trimmer et al., 2016) do not parameterize N_2_O consumption, and other models either consider N_2_O consumption only in suboxic or anoxic waters (Cornejo and Farías, 2012; Babbin et al., 2015) or estimate N_2_O consumption assuming it is constrained by O_2_ concentration (Zamora et al., 2012). Failing to consider the O_2_-independent, non-denitrification N_2_O consumption potential in these O_2_ forcing models might contribute to their uncertainty and the variation among different models. Additionally, the N_2_O consuming organisms have not been fully investigated. Besides denitrifiers and atypical N_2_O consuming bacteria analyzed in this study, other organisms (*Trichodesmium* and *Crocosphaera*) also exhibited N_2_O consuming capacity under laboratory conditions (Farías et al., 2013), suggesting that their significance in the environment warrants further investigation.

## Conclusion

The results described above support two (1 and 3) of the initial hypotheses. (1) Compositions of total and active *nosZ* assemblages were different between the coastal station and the off-shore station mainly due to their dramatic differences of distance to the sediment and to the shore, which are very likely to result in different environmental conditions (i.e., different phytoplankton assemblages, different nutrients and organic matter). (2) The abundances of total and active *nosZ* assemblages in oxygenated seawater were similar to or larger than those in the ODZs, implying the potential for N_2_O consumption even in oxygenated surface water. Atypical *nosZ* archetypes, which may lack a complete denitrification pathway, dominated both total and active *nosZ* assemblages. (3) The total and active *nosZ* assemblages were significantly different from each other. The community composition of the total *nosZ* assemblage showed O_2_ dependence and shifted along depth gradients and environmental gradients associated with depth, but fluorescence, N_2_O and nitrite concentration were significantly correlated with the composition of the transcriptionally active community. We conclude that the difference between active and total *nosZ* assemblages may be related to differential response to environmental conditions by different components of the diverse natural assemblage and that the presence of *nosZ* assemblage in surface waters should be investigated to determine their actual N_2_O reduction capabilities.

## Author Contributions

XS and BW designed the experiments. AJ and BW collected samples. XS and AJ performed experiments. XS analyzed the data. XS and BW wrote the paper.

## Conflict of Interest Statement

The authors declare that the research was conducted in the absence of any commercial or financial relationships that could be construed as a potential conflict of interest.

## Footnotes

1 http://itol.embl.de/
