# Supplementary material for: Community Composition of Nitrous Oxide Consuming Bacteria in the Oxygen Minimum Zone of the Eastern Tropical South Pacific
Source: Front Microbiol. 2017 Jun 28;8:1183. doi: 10.3389/fmicb.2017.01183 (PMC5487485; doi:10.3389/fmicb.2017.01183)
Supplement: Supplementary file 1 [file Data_Sheet_1.PDF]

Supplementary Material

Community composition of nitrous oxide consuming bacteria in the oxygen minimum zone of the Eastern Tropical South Pacific

Xin Sun*, Amal Jayakumar, Bess B. Ward

*** Correspondence:** Xin Sun: xins@princeton.edu

1 Supplementary Figures and Tables

1.1 Supplementary Figures

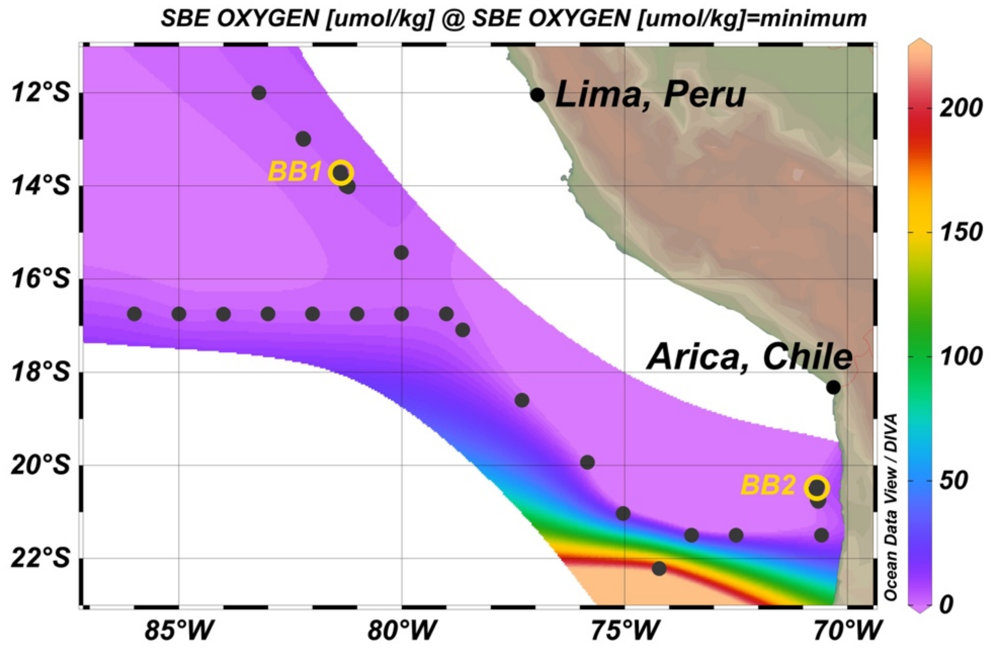

Supplementary Figure 1. Location of sampling stations (BB1 & BB2) and minimum oxygen concentration at the vertical minimum (µmol/kg).

(A)

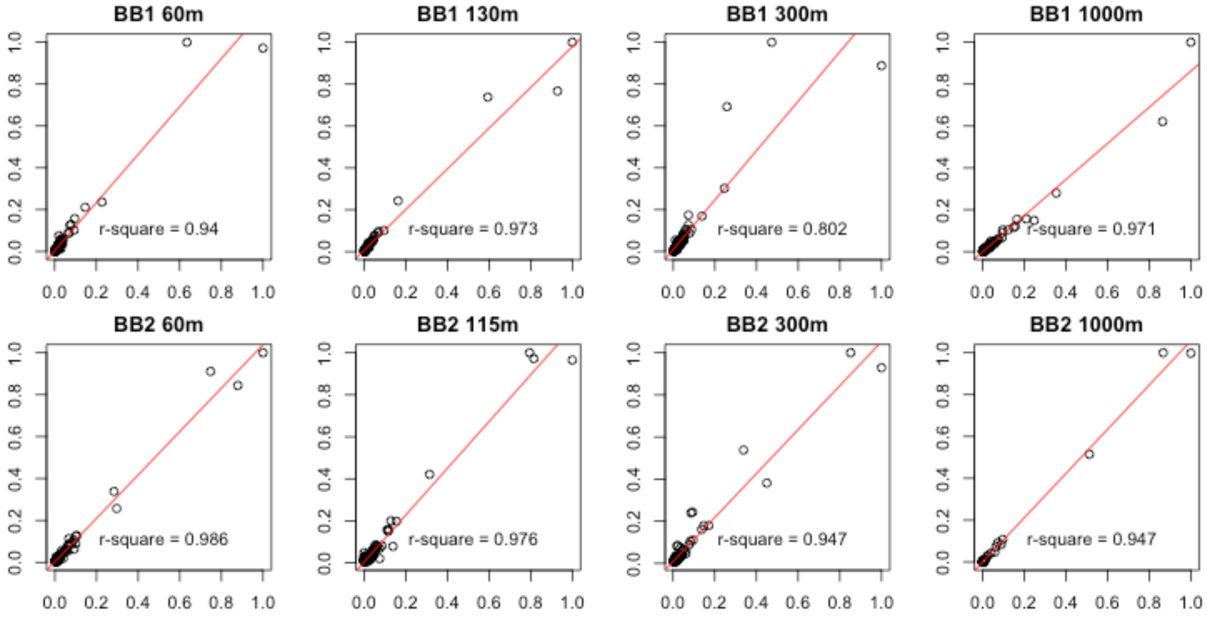

(B)

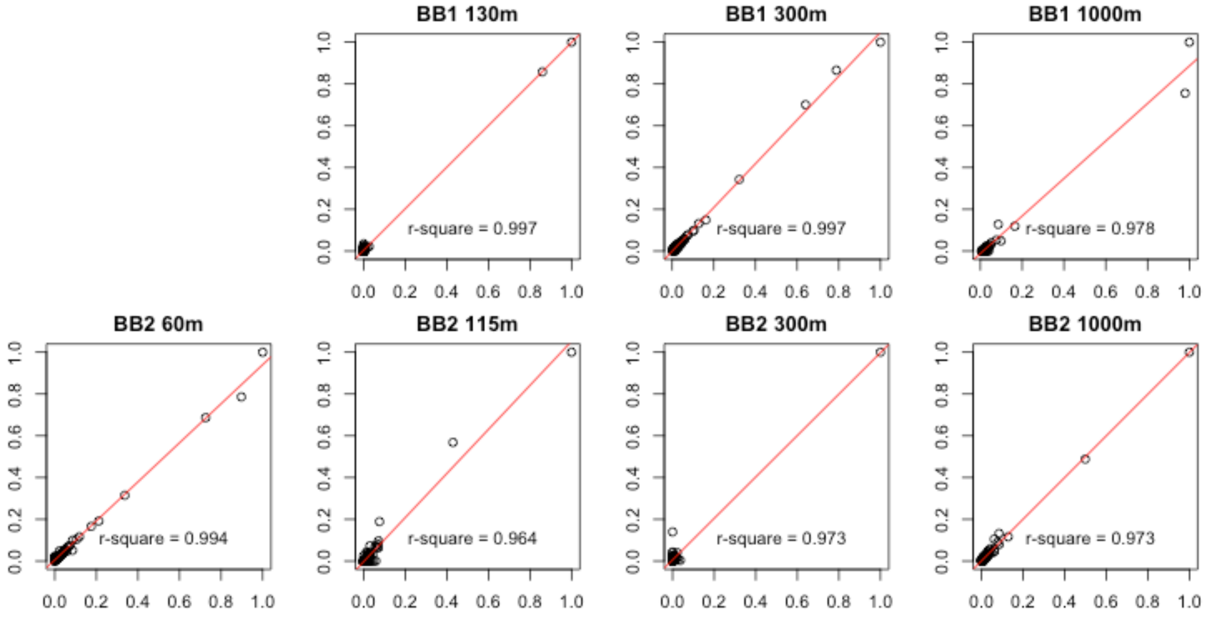

Supplementary Figure 2. Linear regression results of FRn for **(A)** DNA and **(B)** RNA duplicates on microarrays.

1.2 Supplementary Tables

Supplementary Table 1 Environmental variables.

	BB1-60m	BB1-130m	BB1-300m	BB1-1000m	BB2-60m	BB2-115m	BB2-300m	BB2-1000m
**O_2 [µmol/kg]**	209.4	4.2	2.5	54.5	41.9	2.1	2.4	65.2
**NO_3^- [µM]**	3.3	19.9	29.2	44.2	23.3	9.7	21.2	43.5
**NO_2^- [µM]**	0.191	0.035	3.433	0.014	0.083	5.604	6.709	0.003
**NH_4^- [µM]**	0.391	0.063	0.032	0.018	0.099	0.032	0.039	0.121
**N_2O [µmol/kg]**	51.0	12.4	29.5	43.5	90.4	17.9	8.3	42.8
**N***	-6.6	-21.3	-9.7	-3.5	-12.3	-27.0	-14.1	-4.4
**Temperature [^{o}C]**	18.7	13.0	10.5	4.3	13.5	12.9	10.9	4.2
**Salinity [PSU]**	35.4	34.8	34.8	34.5	34.7	34.9	34.8	34.5
**Sigma Theta**	25.4	26.2	26.7	27.4	26.1	26.3	26.6	27.4
**Bot. Depth [m]**	4900	4900	4971	4900	1790	1782	1714	1837
**Pressure [DB]**	59.9	129.9	302.0	1008.3	60.0	115.3	301.6	1006.9
**Fluorescence [mg/m^3]**	0.789	-0.037	-0.059	-0.027	0.301	0.021	-0.029	0.011

Supplementary Table 2 Sequences for *nosZ* probes.

Probe name	Sequence
NosZ1	TGCAAATGGAATATCGACAAAGCCATCCGGAAGTACGCCGGCGAGAAAGTCGACCCCATCGTGCAAAAAC
NosZ2	GTCAAGTGGGATATCGACAAGGCCATCCGGGCCTTCGCCGGTGAAGACGTCGACCCGATTGTCTCAAAGG
NosZ3	GTGAAATGGAACATCGACAAGGCTATCCGCGCTTACGATGGGGAGGATGTTGATCCAATAGTCTCGAAAG
NosZ4	GTGAAGTGGAACATCGACAAGGCAATCCGCGCCTATGCGGGCGAGGATGTCGATCCGATCATTTCCAAGG
NosZ5	GTCAAATGGAACATTGACAAGGCCGTAAAGGCATATGGCGGTGCCGACGTCGATCCGATCATCTCCAAGG
NosZ6	TGCAAGTGGAACGTGGCCGATGCCATCCGCCACTACAACGGTGAGAAGGTGAACTACATCCGCCAGAAGC
NosZ7	TGCAAATGGAACATTGATCTCGCCATTCGCGCTTATGCCGGCGAAAACGTTGATCCGATCGTGCAGAAGA
NosZ8	GCGAAATGGAACATGGAAAAGGCCATTCGCCAGTTCAAAGGCGAAGACGTAGATCCAATTATCGAGAAGA
NosZ9	GTGAAATGGAACATCGACAAGGCGATCCGCGCATATTCGGGCGAAGCGGTCGATCCGATCATCTCGAAAA
NosZ10	GTGAAGTGGAACATCGCCAAGGCGATCGAGGCCTATGGCGGCGCCGATGTCGACCCGATCATCTCCAAGG
NosZ11	GTGAAATGGAACCTTGAAAAGGCGATCCAGCTTTACAATGGTGAGGAAGTGGACCCGATCATCACCAAGC
NosZ12	GCCAAGTGGAACATCGAGATGGCGAAGCGCAAATATGCCGGCGAAAAGGTCGATCCGATCCTCGACAAGC
NosZ13	GTGAAGTGGAACATTGAGAAAGCGGTCCAGCAGTTCCAAGGCGAAGACGTCGATCCAATTATCGAGAAGA
NosZ14	GCGAAATGGAACATCGAAAATGCCATTCGCCACTACAACGGCGAGCAGGTGAACTACATCCGCCAGAAGC
NosZ15	GTCAAGTGGAACATAGACAAGGCCAAGCGCGCGTTCAAGGGCGAGAAGGTCGATCCGATCATCCAGAAGC
NosZ16	GTGAAATGGGACATCGCCAAGGCGGTGCGCCTTTATGCCGGCGAGGAGATCGACCCGATCGTGTCGAAGG
NosZ17	GTGAAATGGAACATCGACAAAGCGATCAGGAAGTATAACGGCGAGGACGTTGACCCGATCATCTCCAAGG
NosZ18	TGCAAATGGAACATTGAAGACGCCAAGCGCGCATTCAAGGGCGAAAAGGTTGATCCGATCCGTCAGAAGC
NosZ19	GTCAAATGGAACCTGGAAGACGCCCGCCGCGCCTACAAGGGCGAGAAGGTCGACTACATCCGACAGAAGC
NosZ20	GTGAAGTGGAACATGGACGAGGCCATCCGCGCCTACAAGGGCGAGAAGGTCAATTACATCAAGCAGAAAC
NosZ21	GTGATGTGGAACATCGAAAAAGCCGTGCGTGCCTATAACGGTGAAAGCGTGGACCCGATCCTGAACAAAC
NosZ22	GTGATGTGGAACATCGAGGATGCGATCCGAGCTCACAACGGCGAGGATGTGGACCCGATCCGCACAAAGC
NosZ23	GTTGTTTGGGACATTGCGAAAGCGATCCAGCTTTATAACGGCGAGGATGTCGACCCCATCATTACCAAAG
NosZ24	TGCAAATGGAACATCGATCTGGCCAGGCGCGCCTACAAGGGCGAGAAGGTCAATCCGATCATCGACAAGC
NosZ25	GTCAAGTGGGATATCGACAAGGCCATCCGGGCCTTCGCCGGTGAAGGCGCGCAGCCGGTGCTCGACCGCA
NosZ26	TGCAGCTGGAACATCGACTTGGCGATTCGAAAGTTCAACGGCGAGGACGTCGATCTAATCCTGGCCAAGA
NosZ27	GTGAAATGGAACATCGAAGAAGCTATCAAAGCGTATTCAGATGAGAATATTGATCCGATTGTGTCCAAGG
NosZ28	GCCAAGTGGAACATCGACGATGGCGAACCGCAATATGCCGGCGAAAAGGTCGATCCGATCCTCTCGAAGC
NosZ29	GTGAAGTGGAACATCCAGAAAGCCATCGATGCCTATGGCGGTGCCGATGTCGATCCGATCCTCGACAAGG
NosZ30	GTCAAGTGGAACATCGACAAGGCCCTGCGCGCCTATGCCGGCGAAGACGTCGATCCGATCGAACAGAAGG
NosZ31	GCCAAGTGGAACATCGAGGACGCGGTGAAGGCCTATTCCAACAAGGAGGCCGCCCCGATCCTCGAGAAGA
NosZ32	GCCAAGTGGAATCTCGATGCGGCAGTGCGCCAATATTCCGGTGAAGACGTCGATCCGATCCTTGCCAAGA
NosZ33	GTCAAATGGGATCTGGAGAAGGCCGTGCGCGCCTATGCCGGCGAGGACGTCGATCCGATCGTCTCCAAGG
NosZ34	GTGAAATGGAACATGGAAAAGGCGATCAAGGCGTTTGCCGGTGAAAAGGTTGACCCGATCATCTCAAAGG
NosZ35	GTAAAGTGGAACATCGATGAGGCCATCCGCGCCTACGCCGGTGAGAAGATCAACCCGATCAAGGACAAGC
NosZ36	GTCAAATGGAACATCGATCTCGCCAAACGAGCCTTTAAGGGCGAGAAGGTCGATCCCATCATCCAGAAGC
NosZ37	GCCAAGTGGAACGTGGAAGAGGCGATTCAGGCCTACCAGGGCAAGAAGGTCAACTACCTGCGCCAGAAGC
NosZ38	GTGAAATGGAACATCGATATGGCGGTCCGCCGCTATGCCGGCGAGGACGTCGACCCGATCCTCTCCAAGA
NosZ39	GTGAAATGGAACATCGCCAAGGCCATCAAGCAATACAACGGTGACGACGTGAATTACCTCATCCAGAAGA
NosZ40	GTGAAATGGAACATCGCCGACGCGATCAAGCACTACAACGGCGAAGAAGTCGATCCGATCATTTCAAAGG
NosZ41	GTAAAATGGAACCTTGAAAAGGCGATCCGTGGTTATAATTGCGAGGAAGTGGACCCGATCATCCAGAAGC
NosZ42	GTGAACTGGAACATCGAGAAGGCTATCCAGGCCTACAACGGCGAGGACGTGGATCCGATCATCCAGAAGC
NosZ43	GTTAAATGGAATATTGACAAGGCCATCAAGGTATTTGCCGGCGAAGACGTCGATCCGATCATATCGAAAG
NosZ44	GCGAAATGGAACATTGAAAATGCCATTCGCCACTACGGCGGCGAGGAAGTGAATCCGATCCTCCCCAAAC
NosZ45	GCGAAATGGAACATGGACAATGCCATCCGCGCCTACAATGGCGAGAGCGTGGACTACATACGCCAGAAAC
NosZ46	GCCAAGTGGAACATCGAGGACAGCGGTGAAAACTATTCCAACAAGGAAGTCGCCCCGATCCTCGAGAAGA
NosZ47	GTGAAGTGGAACATCGCAGATGCGATCAAGCTCTATAACGGTGAAAGCGCCGATCCGATTCTGTTCAAGA
NosZ48	GTGAAGTGGAACCTCGAGGATGCCGTGCGCGCCTTTGCGGGCGAGGACGTGGATCCGATCATCGAAAAGA
NosZ49	GTGAAGTGGAACCTCGAGACGGCAGTGCAGGCCTTCGCCGGCGAGGAGGCGGACCCGATCATCTCGAAGG
NosZ50	GTCAAGTGGAACATGGAGACGGCGATCAAGGCTTTCCAGGGCGACAAGGGCGAGCCGGTGCTCGACCGCA
NosZ51	GCCAAGTGGAATATCCAGAAAGCGGTCGACGCTTACAGCGGCAAGGACGTCGATCCGATTCTGCAGAAGA
NosZ52	GCAAAATGGAATGTCGAAGATGCAATCAAGGCTTACAACGGCGAGAAGGTGAATTACCTGCGCCAGAAAC
NosZ53	TGCAAGTGGAACATGGATGCCGCGTTGCGCCAGTACGCCGGCGAAGACGTCGATCCGATCCTGGCGAAGA
NosZ54	GCCAAATGGAACCTGGAGGCCGCCGTCAGGCAGTATGCCGGCGAGGACGTCGATCCGATCCTCCAGAAAA
NosZ55	GTGAAATGGAACATCGATCTGGCCAAGCGGCTTTACGCGGGCGAAAAGGTCGATCCGATCCTCGACAAAT

NosZ56	TGTAAGTGGAACATCGATGCGGCCCGGCGTGCGTACAAGGGCGAGAAGGTCGACCCGATCCTTCAGAAGC
NosZ57	TGCAAGTGGAACCTGGATCTCGCAAAGCGCGCCTACGCGGGTGAAAAGGTCAATCCGATCATCCAGAAGC
NosZ58	GTGAAGTGGAACATCGAGAAGGCCCGGCAGGCCTACAAGGGCGCCAAGGTCGACCCGATCCTCCAGAAGC
NosZ59	TGCAAATGGAGCCTCGATCTCGCCAAGCGCGCGTTCAAGGGCGAGAAAGTGAACCCGATCATCCAGAAGC
NosZ60	TGCAAATGGAACATCGAGGACGCCAAGCGGGCCTATGCAGGCGAGAAGGTCGATCCCATACGCCACAAGC
NosZ61	GTGAAGTGGAACATCGACAAGGCCAGGCGCGCCTATACGGGCGAGAACGTCTCCCCCATCGTGCAGAAGC
NosZ62	GTCAAGTGGAACATCGACAAGGCCCGTCGCGCCTACCTGGGCGAAAAAGTCGATCCCATCATCCAGAAGC
NosZ63	GTTAAGTGGGACATCGACAAAGCGATCCGTCAGTATGCAGGCGAAGACGTCGATCCAATTCACTCAAAAA
NosZ64	GCCAAGTGGAACATCGCCGACGCGATCGCCGCCCACGACGGCAAGAAGGTCAACTACCTGCGCCAGAAGA
NosZ65	GTGAAGTGGAACATTGCAGATGCCATCAAGCATTACAACGGTGAAGACGTAAACTACCTGCGCCAGAAGC
NosZ66	GTCAAGTGGAACATGGAGGATGCGGTCCGCCACTTCCAGGGCGAGAACGTCAACTACATCCGGCAGAAGT
NosZ67	GCGAAGTGGAATATCGCCGAAGCCATACGCGCTTATAACGGAGAGAAGGTTAACTATATTAAGCAGAAGT
NosZ68	GCCAAATGGAATATTGAAGATGCGATCAAAGCGCATAATGGTGAAAAAGTGAAATATCTTCGCCAGAAAC
NosZ69	GCTAAATGGAATGTTGAAGATGCTATTAAAGCGTACAAAGGCGAAAAAGTTAACTACATCCGTCAAAAAC
NosZ70	GCAAAATGGAATGTAGAAGATGCAATCAAAGCTTACAATGGAGAGAAAGTAAACTATATCCGTCAAAAGC
NosZ71	GCTAAGTGGAATATCCAAGATGCGATCAAGGCCTACAATGGCGAAAAAGTAAATTACTTGCGTCAAAAGC
WNZ1	GTCGAACGTGACGGCCAAGGTCGTTCTCGTGCGCTCGACGATCACCCCAACCGCGATCGAGGTCAACCAG
WNZ2	CAACAAGGTCACCGTCAAGATGATCGCGGTCCGCTCCACGCTGACGCCGACGTCATTCGAGGTGCGCGAC
WNZ3	CAACCGCGTGACGGTGAAGATGGTCGCGGTCCGATCCAGCTTCACGCCCGCCACCTTCGAGGTGCGCGAA
WNZ4	CACAAACGTGACCGTCAAGCTGGTCGCGGTGCGCAGCAGCTTCGAGCCGAACAAGATCGAGGTCAACCGG
WNZ5	CAACAAGGTGATCGCGAAGGTGGTGGAGGTCCGGTCGACCATCACCCCCACCTCGATCGAGGTGAACGAG
WNZ6	AAATACGGTGATCGCCAAAGTGCTGGCTGTACGCAGCACGATTGAACCGACCAAAATCGAAGTCAATCAG
WNZ7	TGGAACGGTAGAGGTAAAGATGGTCGCAGTACGTTCCACACTGCACCCGACAAACGCGGAGGTCAACCGA
WNZ8	CGCCAATGTGACGGCCAAGCTGGTCGCCGTCCGGAGCAGCTTCGAACCGACCAAGCTTGAGGTCAACCAG
WNZ9	CGCTAATGTGACGGTGAAGATGGTCGCCGTCCGCAGCACACTCGAACCGACTTCGATCGAGGTCAACCAG
WNZ10	CAACAAGGTCACCGTCAAGATGATTGCCGTCCGTTCCACATTCACCCCGACGAACTTCGAGGTGCAGGAA
WNZ11	CAACAAGGTCACGGTCAAGATGGTGGCGGTGCGCTCGACCCTGACCCCCACCGACTTCGAGATCAAGGAA
WNZ12	GAGCGAGGTCCTCGCCAGGGTCGTCCTCGTCCGCTCCACGATCACCCCGCAGGCGATCGAGGTCCAGCAG
WNZ13	CAACAAGGTGCTCGCCAAGGTCATCGCCGTGCGATCCACGCTGACGCCGACCTCGATCACCGTGACCGAA
WNZ14	GAGCGAGGTCCTCGTCAAGATGGTCCTCGTCCGGTCGACCATCACTCCGACCGTCATCGAGGTGAACCGG
WNZ15	CGACAAGGTCACGGTCAAGGTGCTCGAGGTGCGCTCGACCATCAGCCCGACCTCGGTGGAGGTGAACGAG
WNZ16	GAGCGACGTGCTCGTCAAGGTGGTGGTGGTCCGCTCGAGCTTCACGCCCTCGCTCATCGAGGTGAACCAG
WNZ17	GAACAAGGTGACGGTGAAGATGATCGAGGTGCGCTCCACCATCACGCCGACCGACTTCGAGGTCAAGGAA
WNZ18	GAACCACGTGACCGTGAAGATGGTCGCAGTGCGTTCCAGCTTCTATCCGCCGACGTTCGAGGTCAACGAG
WNZ19	CAAGAAGGTGCTGATCAAGATGGTGGCCGTGCGCTCGACGCTGTCACCGACCGATTTCTCGGTCGAGGAA
WNZ20	GAGCGAGGTCGTCGTCAAGATCGTCGCGGTGCGCTCCAGCTTCACGCCGTCGGCGGTCGAGGTGAACCAG
WNZ21	CAACGAGGTGCTGGCGAAGGTGGTGGTGGTGCGGTCCAGCATGACGCCGGCGCTCATCGAGGTGAACGAG
WNZ22	CAAGAAGGTGCTGGTCAAGATGGTGGCGGTGCGCTCCACGATCACGCCCACGTCGTTCGAGCTCAGCGCC
WNZ23	CGACCAGGTGATCGCCAAGGTGGTGGCCGTGCGCTCGACGCTGTGGCCCACCTCCATCACGGTCAAGAAG
WNZ24	CAAGAAGGTGCTGGTGAAGATGGTCGCCGTCCGCTCCAGCTTCTCGCCGACCTCGTTCGAGGTGAACGCC
WNZ25	CAAGAAGGTCACGGTGAAACTGGTGGCGGTGCGCTCGACGTTCACGCCATCGAGCTTCGAGGTCGCGGCC
WNZ26	CAACAAGGTCGTCGTCAGGATGGTGGCGGTGAGGTCCACGCTGACGCCCACCTCCTTCGAGATCAAGAAC
WNZ27	CAGCAACGTAACGGTCAAAGCCGTTCTCGTCAGATCGACGATTACGCCCACCTCGTTCGAAGTCCGGCTG
WNZ28	GCGTGTGGAGCGGCCCGAGGCCGACCGGCACCTCCGCGTCCTTGATCGACTCGTACTTCAGGACCGGGAT
WNZ29	GCAGGTGCTCCTGCCGCCGTTCGACTGGGACCTCGGCGACGCCGGCAAGAAGCTGTCGGAGGGCTGGTTC
WNZ30	GCAGGTGGCGCTCCCGCCGTTCGACTGGGATCTGGGCGACGCCGGCAAGCTCGTCTCCGACGGGTGGATG
WNZ31	GTACAACATCGGCCATATCGCTTCCTGTGAAGGCGACACGGTCTCGCCAAAGGGCAAGTGGGTGTTCGCA
WNZ32	ATATTCGCCAGGGCATGCCGTCGCGGCGGAAGGGGATACCGTATCTCCGGACGGCAAATATTTAGTGTCG
WNZ33	AGCAGGCCACCGCAGCGGCAACCGCCGGCAAAGGCAAGATCGTGGGCGGTGCACGGGTCCTCGAACCGAA
WNZ34	AGCAGGCCACCGCGGCCGCCACGGCCGGCAAAGGCAAGATGGTGGGCGGCGTGCAGATGCTCGACCCGAG
WNZ35	AGCACCAGTACACGCCCGGCTTGCCGGCCGTGAACGTCACCGACTTCGTCTCCTGCGGGTTGACGATGAA
WNZ36	AGAAGGCGAAGACGCCGTCGCGATCGGCCACGAACTCGATGGTGGCTGTCTCGCCCGGCTCGATGCTCAG
WNZ37	AGTACGGGTACACGCCGGCCTTGTTGGCTTTGACGGTCAGCGAGGCGGTCTTGCCCGGCTCGATGGACAG
WNZ38	GCTCGTGGCGATGAACAAGCTCCAGAAGGGCCGCGGGCTTAACGTCGGCCCCGAGAAGCCGGAGACGAGC
WNZ39	TCTGGTCGCCGGCAACAAGCTCTCGAAGGGGCGGCACCTCAGCACGGGTCCGTCGCAGCCCGAGAGCAGC
WNZ40	CGTCGTGGCGATGAACAAGATCACGAAGGATCGGTATCTCCCAACGGGCGCTGAGCTGACGCAGTCCGCG
WNZ41	TCTCGTGGCGATGGACAAGATCACCAAGGATCGCTACCTGCCGACGGGCCCCGAGCTCTTCCAGGCGGCA
WNZ42	CCTGGTCGCCATGAACAAGTGGTCGCTCGACCGATTCAATGACGTGGGGCCGCTGCACCCGCAGAACTTC
WNZ43	CCTGGTCGCGCTCAACAAGCTGGCCATCGACCGCTTCAACCCGGTCGGCCCACTGCACCCGCAGAACCAC

Supplementary Table 3 Top five total (DNA) *nosZ* archetypes at each depth.

	Archetypes	Origin of each archetype	FRn %
**BB1-60m**	WNZ21	*Anaeromyxobacter dehalogenans* from soil	24.20%
	WNZ16	uncultured clone from soil	20.10%
	NosZ15	*Achromobacter xylosoxidans* A8 from soil	5.70%
	WNZ36	Complete genome from marine hot spring	4.40%
	WNZ22	uncultured clone from soil	3.10%
		**The sum of the top five archetypes**	**57.50%**
**BB1-130m**	WNZ21	*Anaeromyxobacter dehalogenans* from soil	22.30%
	WNZ16	uncultured clone from soil	18.90%
	NosZ65	*Marinobacter sp.* BSs20148 from marine sediment	14.90%
	NosZ6	uncultured clone from marsh	4.50%
	WNZ43	uncultured clone from waste water treatment sludge	2.20%
		**The sum of the top five archetypes**	**62.90%**
**BB1-300m**	WNZ21	*Anaeromyxobacter dehalogenans* from soil	17.90%
	WNZ16	uncultured clone from soil	13.90%
	NosZ65	*Marinobacter sp.* BSs20148 from marine sediment	9.00%
	NosZ6	uncultured clone from marsh	5.20%
	WNZ43	uncultured clone from waste water treatment sludge	2.90%
		**The sum of the top five archetypes**	**48.90%**
**BB1-1000m**	WNZ21	*Anaeromyxobacter dehalogenans* from soil	21.60%
	WNZ16	uncultured clone from soil	16.10%
	NosZ64	*Rhodanobacter denitrificans* 2APBS1	6.90%
	NosZ14	uncultured clone from marsh	4.30%
	NosZ6	uncultured clone from marsh	4.00%
		**The sum of the top five archetypes**	**52.80%**
**BB2-60m**	WNZ21	*Anaeromyxobacter dehalogenans* from soil	17.40%
	WNZ16	uncultured clone from soil	15.00%
	NosZ65	*Marinobacter sp.* BSs20148 from marine sediment	14.50%
	NosZ6	uncultured clone from marsh	5.40%
	NosZ15	*Achromobacter xylosoxidans* A8 from soil	4.80%
		**The sum of the top five archetypes**	**57.20%**
**BB2-115m**	WNZ21	*Anaeromyxobacter dehalogenans* from soil	14.80%
	WNZ16	uncultured clone from soil	13.50%
	NosZ65	*Marinobacter sp.* BSs20148 from marine sediment	13.50%
	NosZ6	uncultured clone from marsh	5.50%
	WNZ43	uncultured clone from waste water treatment sludge	2.70%
		**The sum of the top five archetypes**	**50.00%**
**BB2-300m**	NosZ65	*Marinobacter sp.* BSs20148 from marine sediment	16.30%
	NosZ6	uncultured clone from marsh	15.60%
	WNZ21	*Anaeromyxobacter dehalogenans* from soil	7.40%
	NosZ14	uncultured clone from marsh	7.00%
	WNZ16	uncultured clone from soil	3.00%
		**The sum of the top five archetypes**	**49.20%**
**BB2-1000m**	NosZ14	uncultured clone from marsh	31.60%
	NosZ6	uncultured clone from marsh	29.50%
	NosZ65	*Marinobacter sp.* BSs20148 from marine sediment	16.20%
	NosZ42	uncultured clone from marsh	3.20%
	WNZ21	*Anaeromyxobacter dehalogenans* from soil	2.70%
		**The sum of the top five archetypes**	**83.30%**

Supplementary Table 4 Top five active (RNA) *nosZ* archetypes at each depth.

	Archetypes	Origin of each archetype	FRn %
**BB1-130m**	WNZ21	*Anaeromyxobacter dehalogenans* from soil	47.2%
	WNZ16	uncultured clone from soil	40.6%
	WNZ15	*Marinobacter sp.* BSs20148 from marine sediment	1.1%
	WNZ22	uncultured clone from soil	0.8%
	NosZ35	*Achromobacter cycloclastes from soil*	0.8%
		**The sum of the top five archetypes**	**90.5%**
**BB1-300m**	WNZ21	*Anaeromyxobacter dehalogenans* from soil	19.0%
	WNZ16	uncultured clone from soil	15.7%
	WNZ28	*Anaeromyxobacter dehalogenans*	6.3%
	WNZ1	uncultured clone from soil	12.8%
	NosZ6	uncultured clone from marsh	3.0%
		**The sum of the top five archetypes**	**56.8%**
**BB1-1000m**	WNZ21	*Anaeromyxobacter dehalogenans* from soil	30.6%
	WNZ16	uncultured clone from soil	26.5%
	WNZ28	*Anaeromyxobacter dehalogenans*	4.3%
	WNZ1	uncultured clone from soil	2.3%
	NosZ11	uncultured clone from marsh	3.3%
		**The sum of the top five archetypes**	**66.9%**
**BB2-60m**	WNZ21	*Anaeromyxobacter dehalogenans* from soil	17.8%
	WNZ16	uncultured clone from soil	15.0%
	NosZ15	*Achromobacter xylosoxidans* A8 from soil	12.6%
	NosZ67	*Hahella chejuensis*	5.8%
	WNZ9	uncultured clone from soil	3.6%
		**The sum of the top five archetypes**	**54.7%**
**BB2-115m**	WNZ21	*Anaeromyxobacter dehalogenans* from soil	34.3%
	WNZ16	uncultured clone from soil	17.1%
	WNZ34	uncultured clone from soil	4.5%
	NosZ35	*Achromobacter cycloclastes from soil*	2.9%
	WNZ22	uncultured clone from soil	2.6%
		**The sum of the top five archetypes**	**61.4%**
**BB2-300m**	WNZ21	*Anaeromyxobacter dehalogenans* from soil	66.0%
	NosZ36	*Bradyrhizobium japonicum*	4.6%
	WNZ16	uncultured clone from soil	2.1%
	WNZ11	uncultured clone from fluidized bed reactor treating groundwater	1.4%
	NosZ24	*Rhodopseudomonas palustris*	1.3%
		**The sum of the top five archetypes**	**75.4%**
**BB2-1000m**	WNZ21	*Anaeromyxobacter dehalogenans* from soil	24.0%
	WNZ16	uncultured clone from soil	11.9%
	WNZ22	uncultured clone from soil	2.9%
	WNZ43	uncultured clone from waste water treatment sludge	2.6%
	WNZ34	uncultured clone from soil	2.0%
		**The sum of the top five archetypes**	**43.5%**
